# Case of Hydrophobia

**Published:** 1867-07

**Authors:** Daniel T. Nelson

**Affiliations:** Prof. of Physiology and Histology


### SELECTIONS.

---

### *Case of Hydrophobia.* By Daniel T. Nelson, Prof. of Physiology and Histology.

T. P. A., a bright, intelligent boy of 15 years, was bitten by the pet family dog on the morning of Nov. 27th, 1866. The dog had shown signs of illness for several days, was irritable, was frequently biting his tail, which seemed covered with some kind of an eruption at its extremity, whether the cause or result of the biting, not known. Character of the eruption, whether vesicular or pustular, not known. Did not eat well, but took *some* food and *drinks.* Drank water the day he bit the patient. Did not froth at the mouth, or show any unusual symptoms except those mentioned, and these were not considered by the family of any consequence, as the dog had had three such attacks before—one during each of the three previous winters. The only noticeable feature of this attack was the time of its occurrence, earlier than usual in the season. The morning on which he bit the patient, he seemed better, and ate a cracker which he gave him. It was while feeding him with this that he snapped at him, apparently without provocation, inflicted a crescent shaped wound an inch and a half long in the left cheek, extending from the mouth, upwards and outwards, passing through the whole substance of the cheek. From the previous history of the dog, having had three attacks apparently in all respects similar, nothing was thought of the wound at the time. It was simply washed and drawn together by adhesive straps, when it healed kindly.

During the day, after biting the patient, the dog presented no different symptoms, though he was not allowed to enter the house on account of his conduct in the morning. A few days after he was found dead, whether killed or not, not known.

No further trouble of any kind was experienced by the patient from the wound until Tuesday, Feb. 26, 1867, when, on coming in from play, snow balling, etc., he complained of "not feeling well;" throat a little sore; "that he had taken cold;" headache. Slept pretty well; sleep disturbed by dreams of fighting, etc.

*Wednesday, Feb. 27th.*—Did not get up in the morning; sleepy; no appetite; thirsty; drank lemonade freely; throat no better.

A homœopathic physician was called, who is said to have treated him for diphtheria. About 12, midnight, began to complain of an uneasy feeling—a pricking sensation—about the cicatrix on the cheek. About the same time, also, slight convulsive movements were noticed in the voluntary muscles. Did not sleep.

*Thursday morning, Feb. 28th.*—Convulsive movements more decided, and easily excited; occur spontaneously every hour, increasing in severity and frequency during the morning. Some difficulty in swallowing; speaks rapidly and loud, (latter, perhaps, on account of slight deafness of mother.)

*February* 28, 3 *P. M.*—I first saw the patient; found him sane, but restless; eyes rather wild and staring; pupil somewhat dilated; tongue slightly furred; protruded spasmodically, and with difficulty; respiration labored; pulse quick; sufficient strength; heart's action accelerated; fluttering; face flushed; countenance excited; skin moist, natural; extremities extended, or moving spasmodically; cannot speak on account of spasmodic action of laryngeal muscles; complains of sense of suffocation in throat and chest; want of sleep and thirst; water and all fluids immediately rejected by sudden and powerful contraction of muscles of throat and chest; ice and all solids spasmodically seized by the mouth, and at length swallowed, but with difficulty. Any attempts at moving the body, swallowing fluids, or speaking, produce tetanic spasms of most of the voluntary muscles; opisthotonos, with strongest convulsions; bowels moved Tuesday; urine passed. Thursday, P. M., 3 o'clock.

*Treatment*, 3.15 *P. M.*—Atropine, gr. 1-25. 3.30 *P. M.* Applied ice bag to whole length of spine. 3.45 *P. M.* Feels better; swallows solids, and breathes with less difficulty; had infus. tobacco made, ℨ iij. to Oj., and boiled to ℨ viij. 4 *P. M.*—Atropine, gr. 1-50, infus. tobacco, ℨ ij., made into bolus, with common flour; swallowed without much difficulty; ice as desired. 4.30 *P. M.*—Convulsive movements again on the slightest exertion, but not so strong.

Atropine, gr. 1-50; too restless to keep ice bag to spine; pupils well from atropine; delirium; face more flushed and eyes injected; breathes easy, and talks freely; desires to leave the room. 5 *P. M.*—Infus. tobacco ℥ ss., in bolus; vomited; ordered to be kept quiet. 5.30 *P. M.*—Henry M. Lyman, M. D., called in counsel; delirium; more quiet; pupils well dilated; pulse 130, and losing strength; patient seems exhausted; lies upon back, head thrown back (ophisthotonos.) No change in plan of treatment deemed advisable; no light or attendant allowed in the room; perfect quiet enjoined. 7.30 *P. M.*—Atropine, gr. 1-50. 8.30 *P. M.*—Atropine, gr. 1-50. 9.30 *P. M.*—Ol. tobaci gtt. j., instead of infus. on bread pill. 10 *P. M.*—Failing rapidly; respiration labored; not conscious. 10.30 *P. M.*—Opisthotonos increasing, as also general convulsions; respiration more labored; white, frothy mucus fills the mouth for the first time; saliva not noticeably increased or changed in character before; chloroform given by inhalation; muscles of chest relax, and respiration more easy; general convulsions also less marked.

Kept sufficiently under its influence to quiet the tetanic spasms until death, 11.30 P. M.; died very quietly. At 11 P. M. the white mucus in the mouth became of a rusty hue.

*Remarks.*—The history of this dog has suggested to my mind the following queries:—(1.) Had this dog had hydrophobia three times before, and recovered, not extending the disease by happening not to bite any one? Or (2) was this a severe attack, proving fatal at last, (as it is not known but the dog died of the disease.) But he did not present many of the supposed symptoms of the disease? Or (3) are there other diseases to which the canine race is liable, during the presence of which the bite of the animal will produce rabies in the human subject?

*Indications for Treatment.*—Without theorizing about the materia morbi, several indications for treatment are plainly presented. 1. To quiet the irritability of the nervous system, particularly of the cord. 2. To prevent reflex action. 3. Support the strength. To meet the 1st, the infus. tobacco was given till the ol. tobaci could be procured, nicotina not being in this market.

Tobacco in some form was suggested, from its seeming efficacy in tetanus, which also affects the cord. But how shall we say it acts? Does it act directly upon the nervous tissue, paralysing it, without particularly affecting others. The ice bag was applied to the spine on the supposition that the cord was hyperæmic, from its excessive irritability, and in this way it was hoped the engorged vessels could be made to contract, and thus the irritability reduced. If this be true, that the cord is in a hyperæmic state, might not several other remedies be used with success—drugs which are said to act upon the muscular coat of the arteries—such as aconite, ergot, turpentine, and perhaps tr. chld. iron and electricity—a strong current—and the bromide of potassium, recently recommended? Does this act in the same way? Will some or any of these drugs act upon vessels in the cord, and not upon vessels elsewhere?

But the second indication suggested was to prevent reflex action. On the authority of Dr. Brown-Sequard, belladona in the form of its active principle, atropine, was used. And is its action really like tabacco, to produce paresis of nervous tissue, acting upon both elements of that tissue, or does it act only upon the fibrous motion, and thus prevent the conveyance of impressions? With this in view, Dr. Brown-Sequard advises the removal of an inch or two of the nerve connecting the wound with the cord. The 3d indication was in this case toward its close, difficult to meet, all fluids being rejected by the mouth, and enemas were not tolerated till late, perhaps too late to be of service. Animal broths, and all nutritious and easily digested articles of food, would seem to be indicated, given by the mouth if we can, by the rectum if we must.—*Chicago Medical Examiner.*

